# TaSYP71, a Qc-SNARE, Contributes to Wheat Resistance against *Puccinia striiformis* f. sp. *tritici*

**DOI:** 10.3389/fpls.2016.00544

**Published:** 2016-04-21

**Authors:** Minjie Liu, Yan Peng, Huayi Li, Lin Deng, Xiaojie Wang, Zhensheng Kang

**Affiliations:** State Key Laboratory of Crop Stress Biology for Arid Areas and College of Plant Protection, Northwest A&F UniversityXiangyang, China

**Keywords:** *Puccinia striiformis* f. sp. *tritici*, wheat, SNARE, plasma membrane, virus-induced gene silencing, resistance, H_2_O_2_ tolerance

## Abstract

*N*-ethylmaleimide-sensitive factor attachment protein receptors (SNAREs) are involved in plant resistance; however, the role of SYP71 in the regulation of plant–pathogen interactions is not well known. In this study, we characterized a plant-specific SNARE in wheat, TaSYP71, which contains a Qc-SNARE domain. Three homologs are localized on chromosome 1AL, 1BL, and 1DL. Using *Agrobacterium-*mediated transient expression, TaSYP71 was localized to the plasma membrane in *Nicotiana benthamiana*. Quantitative real-time PCR assays revealed that *TaSYP71* homologs was induced by NaCl, H_2_O_2_ stress and infection by virulent and avirulent *Puccinia striiformis* f. sp. *tritici* (*Pst*) isolates. Heterologous expression of TaSYP71 in *Schizosaccharomyces pombe* elevated tolerance to H_2_O_2_. Meanwhile, H_2_O_2_ scavenging gene (*TaCAT*) was downregulated in *TaSYP71* silenced plants treated by H_2_O_2_ compared to that in control, which indicated that *TaSYP71* enhanced tolerance to H_2_O_2_ stress possibly by influencing the expression of *TaCAT* to remove the excessive H_2_O_2_ accumulation. When *TaSYP71* homologs were all silenced in wheat by the virus-induced gene silencing system, wheat plants were more susceptible to *Pst*, with larger infection area and more haustoria number, but the necrotic area of wheat mesophyll cells were larger, one possible explanation that minor contribution of resistance to *Pst* was insufficient to hinder pathogen extension when *TaSYP71* were silenced, and the necrotic area was enlarged accompanied with the pathogen growth. Of course, later cell death could not be excluded. In addition, the expression of pathogenesis-related genes were down-regulated in *TaSYP71* silenced wheat plants. These results together suggest that TaSYP71 play a positive role in wheat defense against *Pst*.

## Introduction

Eukaryotes have evolved a specialized class of proteins, the soluble *N*-ethylmaleimide-sensitive factor attachment protein receptors (SNAREs), that functions as mediators of vesicle membrane fusion with specific organelles. Based on the amino acid residues (glutamine or arginine) in the center of the SNARE motif, SNAREs can be grouped as Q- and R-SNAREs. Generally, Q-SNAREs localize to the membrane of target organelles (t-SNAREs), whereas R-SNAREs localize to the transport vesicles (v-SNAREs). Three distinct t-SNAREs and one v-SNARE form a four-helix hetero-oligomeric complex to mediate membrane fusion between vesicles and target membranes, including vesicles, organelles of the endomembrane system, and the plasma membrane (PM). Based on their positions within the assembled four-helix bundle, Q-SNAREs can be classified as Qa-SNARE motifs (occupy the syntaxin position), Qb-SNARE (SNAP-25 N-terminal) and Qc-SNARE (SNAP-25 C-terminal) (Bock et al., 2001).

Recent findings indicate that SNARE functions in plants resistance pathogen against various pathogens (Inada and Ueda, 2014). *Arabidopsis thaliana* syntaxin (At)SYP121 (PEN1, encoding a Qa-SNARE) and its barley (*Hordeum vulgare*) ortholog, HvSYP121 (ROR2), contribute to either non-host resistance or basal penetration resistance against barley powdery mildew (*Blumeria graminis* f. sp. *hordei*) by cell wall reinforcements to avoid penetration (Collins et al., 2003). AtSNAP33 (Qa + Qb-SNARE) forms a ternary SNARE complex with AtSYP121 and AtVAMP721/722 at the PM that is necessary for pre-invasive immune responses in barley and *Arabidopsis* (Kwon et al., 2008). Tobacco (*N. tabacum*) PM SNARE SYP132 has been shown to contribute to defense against bacterial pathogens by mediating the secretion of pathogenesis-related protein 1 (Kalde et al., 2007). Golgi SNARE AtMEMB12 is targeted by miR393b^∗^ and regulates the exocytosis of an antimicrobial pathogenesis-related protein, PR1 (Zhang et al., 2011). AtSYP4 proteins localized to the trans-Golgi network (TGN) contribute to extracellular resistance against fungal pathogens and protect the chloroplasts from salicylic acid-dependent biotic stress (Uemura et al., 2012). In wheat (*Triticum aestivum*), knocking down *TaNPSN11* (Qb-SNARE) expression reduced the resistance of wheat to an avirulent isolate of *Puccinia striiformis* f. sp. *tritici* (*Pst*) (Wang et al., 2014).

SYP7 is one of the SNARE subfamilies unique to plants. The SYP7 subfamily belongs to the Qc-SNAREs and contains SYP71, SYP72, and SYP73. SYP71 is the most studied member of this family. The *Lotus japonicus* SYP71 expressed in vascular tissues has been shown to be involved in symbiotic nitrogen fixation with rhizobia (Hakoyama et al., 2012). Recently, SYP71 has been reported to participate in plant defense against various pathogens. *Arabidopsis* SYP71 is essential for successful viral infection by mediating the fusion of the turnip mosaic potyvirus (TuMV)-induced 6K2 vesicles with chloroplasts during TuMV infection (Wei et al., 2013). Overexpression of OsSYP71 in rice enhanced tolerance to oxidative stress and resistance to rice blast (Bao et al., 2012). However, the exact role of the SYP7 family in defense against biotrophic obligate fungi is limited known.

Wheat stripe rust, caused by *P. striiformis* f. sp. *tritici*, is a devastating worldwide disease. The fungus is strictly biotrophic and cannot survive without the host plant, making it difficult to study the interaction between wheat and *Pst*. In addition, the complex hexaploid nature of the wheat genome makes genetic and functional analyses extremely challenging. In this study, we isolated a wheat SYP71 homolog fragment from the incompatible cDNA library previously constructed by our laboratory, implying a possible role in *Pst* resistance. To find out whether TaSYP71 is involved in wheat resistance, we analyzed its expression patterns under various stresses. Overexpression of TaSYP71 in fission yeast enhanced the ability of the yeast to survive in hydrogen peroxide. Knocking down the expression of *TaSYP71* by a virus-induced gene silencing (VIGS) system reduced the resistance of wheat to CYR23. Therefore, we demonstrated that TaSYP71 plays a positive role in plant resistance possibly through influencing H_2_O_2_ signaling pathways.

## Materials and Methods

### Plant Materials, *Pst* Isolates, and Chemical Treatments

*Triticum aestivum* cultivar Suwon 11 containing the *YrSu* resistance gene was grown at 16°C with a 16 h photoperiod. *N. benthamiana*, which was used for transient expression, was kept at 25°C with a light regime of 16 h light/8 h darkness. *P. striiformis* f. sp. *tritici* (*Pst*) pathotypes CYR23 (avirulent to Suwon 11) and CYR31 (virulent to Suwon 11) were used in this study for wheat and *Pst* interaction assays. Inoculation of *Pst* was performed as described (Kang and Li, 1984).

To study expression levels of *TaSYP71*, wheat leaves were infected with CYR31 or CYR23, and leaf tissues were then sampled at 0, 6, 12, 24, 48, 72, and 120 h post-inoculation (hpi) based on the histological study of the interactions between Suwon11 and CYR23 or CYR31 (Wang et al., 2007). Parallel mock-inoculated control plants were brushed with sterile water. Three biological replicates were performed independently for each assay.

To study expression levels of *TaSYP71* following various chemicals and stress elicitors, wheat leaves were sampled at 0, 6, 12, 24, 48 h post-treatment (hpt). For high salinity and H_2_O_2_ treatment, wheat seedlings were removed from soil, and their roots were soaked in 200 mM NaCl and 10 mM H_2_O_2_, respectively. Osmotic stress was imposed by application of 20% polyethylene glycol 6000 (PEG 6000) in the nutrient solution. All samples were immediately frozen in liquid nitrogen and stored at -80°C. Three biological replicates were performed independently for each assay.

### RNA Extraction, cDNA Synthesis and qRT-PCR Analysis

Total RNA was extracted using the RNeasy Plant Mini Kit (Qiagen) and treated with DNase I according to the manufacturer’s instructions. RNA was reverse transcribed into cDNA with an oligo(dT)_18_ primer using an RT-PCR system (Promega, Madison, WI, USA). The expression patterns of *TaSYP71* under different conditions as described above were detected by qRT-PCR following the procedure previously described (Wang et al., 2009) using a 7500 Real-Time PCR System (Applied Biosystems, Foster City, CA, USA). The wheat elongation factor *TaEF-1a* gene (GenBank accession no. Q03033) was used as the internal reference for all qRT-PCR assays. The relative transcript levels of the pathogenesis-related (PR) protein genes (*TaPR1*, AAK60565; *TaPR2*, DQ090946; and *TaPR5*, FG618781), and reactive oxygen species- scavenging genes (catalase, *TaCAT*, X94352) were also confirmed using qRT-PCR. Primers used in these assays were listed in **Supplementary Table S1**. The comparative 2^-ΔΔCT^ method was used to quantify relative gene expression (Livak and Schmittgen, 2001). Three biological replicates were performed independently.

### Cloning of *TaSYP71* and Sequence Analyses

Specific primers for *TaSYP71* were designed based on the sequence from the cDNA library of the wheat-*Pst* incompatible interaction constructed by our laboratory (Wang et al., 2008). The fragment was cloned into the pMD18-T simple vector and sequenced.

The ORF of *TaSYP71* was aligned with the *T. aestivum* cv. Chinese Spring genome using the International Wheat Genome Sequencing Consortium^1^. The domain structure of the TaSYP71 protein was analyzed using InterProScan (Jones et al., 2014). TMHMM 3.0 was used for transmembrane domain prediction (Krogh et al., 2001). Multiple sequence alignment was performed, and a neighbor joining tree was created using Clustal W (Larkin et al., 2007) and MEGA 6 (Tamura et al., 2013), respectively.

### Subcellular Localization of TaSYP71 in *N. benthamiana*

In the subcellular localization assay, the pCAMBIA1302: TaSYP71-eGFP fusion vector was constructed. pCAMBIA1302: eGFP was used as a control. These two constructs were separately introduced into the *Agrobacterium tumefaciens* strain GV3101 by electroporation. Transformants were selected using kanamycin (50 μg ml^-1^) and rifampicin (20 μg ml^-1^). For infiltration of leaves, recombinant strains of *A. tumefaciens* were grown in LB medium with proper antibiotics to late-log phase, collected, suspended in an infiltration media (10 mM MgCl_2_, 10 mM MES, pH 5.6 and 150 mM acetosyringone), and then maintained at room temperature for 1–3 h before infiltration. *A. tumefaciens* suspensions were infiltrated at an OD_600_ of 0.8 into leaves of 4- to 6-week-old *N. benthamiana* plants using a syringe without a needle. Tissue samples were harvested at 2 or 3 days after infiltration from the infiltration area and directly imaged with an Olympus BX-51 microscope (Olympus Corporation, Japan). Every experiment was repeated at least three times, with each assay consisting of at least three tobacco plants.

### Overexpression of TaSYP71 in *Schizosaccharomyces pombe*

In the overexpression assay, pREP3x: *TaSYP71* was constructed as described (He et al., 1997). The pREP3x: TaSYP71 and pREP3x empty vector were transformed into *S. pombe* by electroporation. Thiamine was used as the repressor of the nmt promoter in the pREP3x vector at a concentration of 2 μg/mL. For assessment of yeast cell sensitivity to H_2_O_2_, cells were removed from logarithmic cultures after 24 h of growth, collected by centrifugation, washed twice with sterile distilled water and finally diluted to densities of OD_600_ = 0.2 with leucine dropout medium containing 20 mM H_2_O_2_. The incubated fission yeast cells were sampled at 4, 8, 12, 16, 20, 24, 28, and 32 h pi. Fission yeast cell growth was also assayed on yeast solid media plates (-leu dropout) with 0, 20, and 60 mM H_2_O_2_ in inducing (without thiamine) or repressing (thiamine) medium. Photos were taken 3–5 days later. Three biological replicates were performed for each assay.

For the western blot analysis, protein was extracted from *S. pombe* as described previously (Moreno et al., 1991). Protein was then separated on a 12% SDS-polyacrylamide gel (SDS-PAGE). The protein was subsequently transferred onto a nitrocellulose membrane using a Semi-Phor Semi-Dry Transfer Unit. The immuno-blot analysis was conducted using the monoclonal antibody raised against GFP (Roche) as the primary antibody and goat anti-mouse IgG-peroxidase conjugate (Sigma–Aldrich, Saint Louis, MO, USA) as the secondary antibody. The immuno-reactivity was detected using an ECL Western Blotting Substrate kit (Thermo scientific, Meridian, Rockford, USA) and photographed.

### BSMV-Mediated *TaSYP71* Gene Silencing

The VIGS system is an effective reverse genetics tool in wheat. The plasmids used for gene silencing were constructed as described by Holzberg et al. (2002). To guarantee the specificity of gene silencing, we searched for silencing fragments that showed the lowest sequence similarities with other genes. Possible silencing off-target effects of these VIGS constructs were tested by si-Fi software (version 3.2) as previously described (Nowara et al., 2010). Based on the criteria described above, a 147-bp cDNA fragment containing part of the 5′ untranslated region and part of the coding sequence and a 126-bp fragment derived from the partial coding sequence to the 3′ untranslated region were used to construct the recombinant *TaSYP71-1* and *TaSYP71-2* plasmids, respectively. Primers used are listed in **Supplementary Table S1**.

Infectious BSMV RNA was prepared from each linearized plasmid by *in vitro* transcription using a high-yield capped T7 transcription kit (mMESSAGE mMACHINE; Ambion). Three independent sets of wheat plants were used. The second leaves of two-leaf wheat seedlings were inoculated with BSMV transcripts. The BSMV inoculum was made by combining an equimolar ratio of α, β, and γ transcripts with excess inoculation buffer containing a wounding agent (Fes buffer) as previously described (Holzberg et al., 2002). The mock control was inoculated with 1× Fes buffer. BSMV: *TaPDS* and BSMV: γ empty vector were used as controls for the BSMV infection. BSMV-treated wheat plants were kept in a cultivation chamber at 25 ± 2°C. When the virus phenotype was observed (∼10 days after BSMV treatment), the fourth leaves of these plants were inoculated with fresh urediniospores of CYR23. The fourth leaves were sampled at 0, 24, 48, and 120 h p-i for histological observation and RNA isolation. The silencing efficiency for each of the BSMV constructs compared with BSMV: γ was examined by qRT-PCR. The phenotypes of the fourth leaves were observed and photographed 14 days after *Pst* inoculation.

### Histological Observation of Fungal Growth and Host Response

Samples leaves were fixed and cleared as described Wang et al. (2007). For microscopic observations, leaf segments were kept in 50% glycerol and examined using differential interference contrast microscopy. The germinated urediospores enter into the host leaf tissue through stomata and form substomatal vesicle. Infection hyphae arising from the substomatal vesicle attempt to penetrate mesophyll cell wall and form haustoria for sustaining further fungal growth. Only infection sites with substomatal vesicles were considered for assessment. The autofluorescence of attacked mesophyll cells was captured using Olympus BX51 fluorescence microscope. H_2_O_2_ accumulation in mesophyll cells was stained by DAB as described (Thordal-Christensen et al., 1997). The area of necrotic cells and DAB stained H_2_O_2_ accumulation cells were measured by DP-BSW program (Olympus) connected with the microscope which can measure the area of closed polygon.

Fungal structures were then specifically stained using wheat germ agglutinin conjugated to Alexa-488 (Invitrogen) as described (Ayliffe et al., 2011). Stained tissue was examined under blue light excitation (excitation wavelength 450–480 nm, emission wavelength 515 nm). The *Pst* hyphae were observed using an Olympus BX-51 microscope, the number of branches and haustoria number were counted, the lengths and the infection area (indicating the ability of fungal expansion) were calculated by DP-BSW software.

## Results

### *TaSYP71* Cloning and Sequence Analysis

A cDNA fragment (938 bp) with a SNARE domain was first isolated from the wheat-*Pst* incompatible cDNA library constructed by our laboratory. The complete open reading frame (ORF) was obtained from cDNA of wheat cultivar Suwon11 infected by CYR23. This gene shared high similarity with *SYP71*, so we designated it as *TaSYP71* (GenBank accession no. KF683945.1). The predicted ORF length of *TaSYP71* is 813 bp, encoding a protein of 270 amino acid residues, with a molecular weight of 30.03 kDa.

Sequence alignment with the *Triticum aestivum* cv. Chinese Spring genome sequence from the UGRI database showed that the sequence was identical with the sequence on long arm of chromosomes 1A. Two other homologs were located on long arm of chromosomes 1B and 1D. The first 154 bp of the ORF was missing in chromosome 1D (**Supplementary Figure S1A**). Only one amino acid residue variation was found between chromosomes 1A and 1B (**Supplementary Figure S1B**), although there were nine nucleotide variations in the ORF of the two copies (**Supplementary Figure S1A**). In addition, only few nucleotide variations were found in 5′ and 3′ UTR region (**Supplementary Figure S1A**).

**FIGURE 1:**
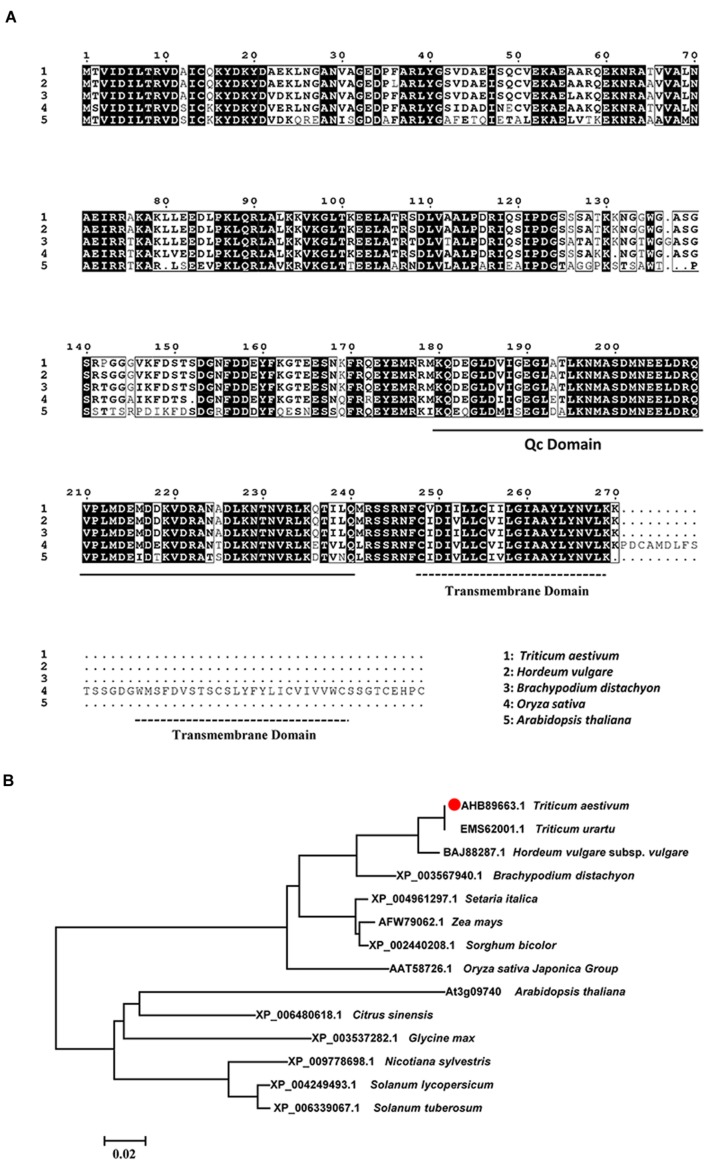
**Multiple sequence alignment and phylogenetic analysis of *TaSYP71* and its homologs from other species.** **(A)** Multiple sequence alignment of amino acids. Identical amino acid residues are shaded in black. Black underline indicates the Qc-SNARE motif, and dotted lines indicate the transmembrane domain conserved in the SYP71 family. **(B)** Phylogenetic analysis of *TaSYP71* and homologs in other plant species. Multiple sequence alignments and the neighbor joining tree were created using the MUSCLE method by MEGA 6.0.

As predicted by TMHMM 3.0, a transmembrane helix located at the C-terminal of TaSYP71. The structural analysis of the TaSYP71 protein showed a Qc-SNARE motif located adjacent to the C-terminal transmembrane anchor. A multiple sequence alignment of TaSYP71 with homologs from other species showed the highest similarity with HvSYP71 (97.78%) in *Hordeum vulgare*. However, TaSYP71 had only 66.67% identity with AtSYP71 in *Arabidopsis* (**Figure 1A**). Phylogenetic analysis revealed that TaSYP71 and its homologs from monocotyledonous were clustered into one large clade in which HvSYP71 showed the highest similarity to TaSYP71. Homologs from dicotyledonous were clustered into another large clade (**Figure 1B**).

### TaSYP71 Was Localized to the Plasma Membrane by Transient Expression in Tobacco

**FIGURE 2:**
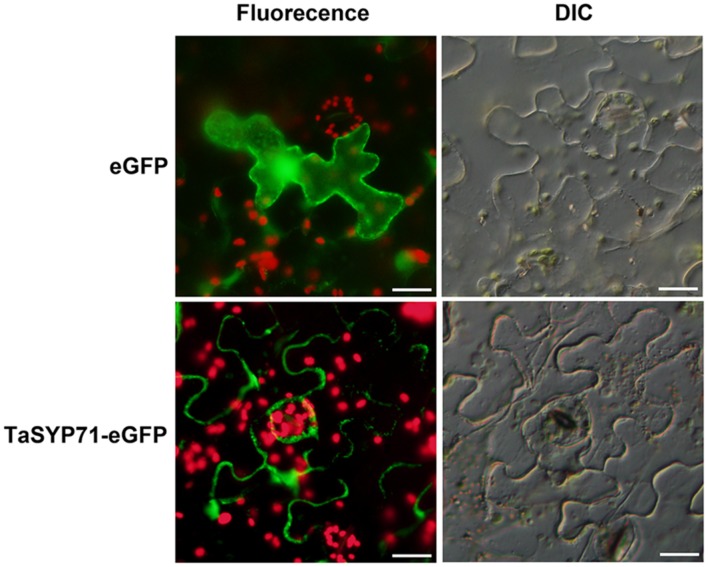
**Subcellular localization of TaSYP71 in *Nicotiana benthamiana* (tobacco).** Enhanced green fluorescent protein (eGFP) (control) and the TaSYP71-eGFP fusion protein were expressed in *N. benthamiana* by transient agro-infiltration assays. The green fluorescence was observed with fluorescence microscopy. The corresponding cell morphology was observed with a differential interference contrast microscope (DIC). Bar = 20 μm.

Previous studies of SYP71 in other organisms have shown that SYP proteins are mainly located in the PM or endoplasmic reticulum membrane. To determine the subcellular localization of wheat SYP71, a TaSYP71-eGFP fusion protein was expressed in *N. benthamiana* using *A. tumefaciens* infiltration. Observation by fluorescence microscopy revealed that, in contrast to eGFP alone, which exhibited fluorescence in both the cytoplasm and the nucleus, TaSYP71-eGFP was restricted to the PM (**Figure 2**).

### *TaSYP71* is Upregulated Following NaCl, H_2_O_2_ and *Pst* Treatment

**FIGURE 3:**
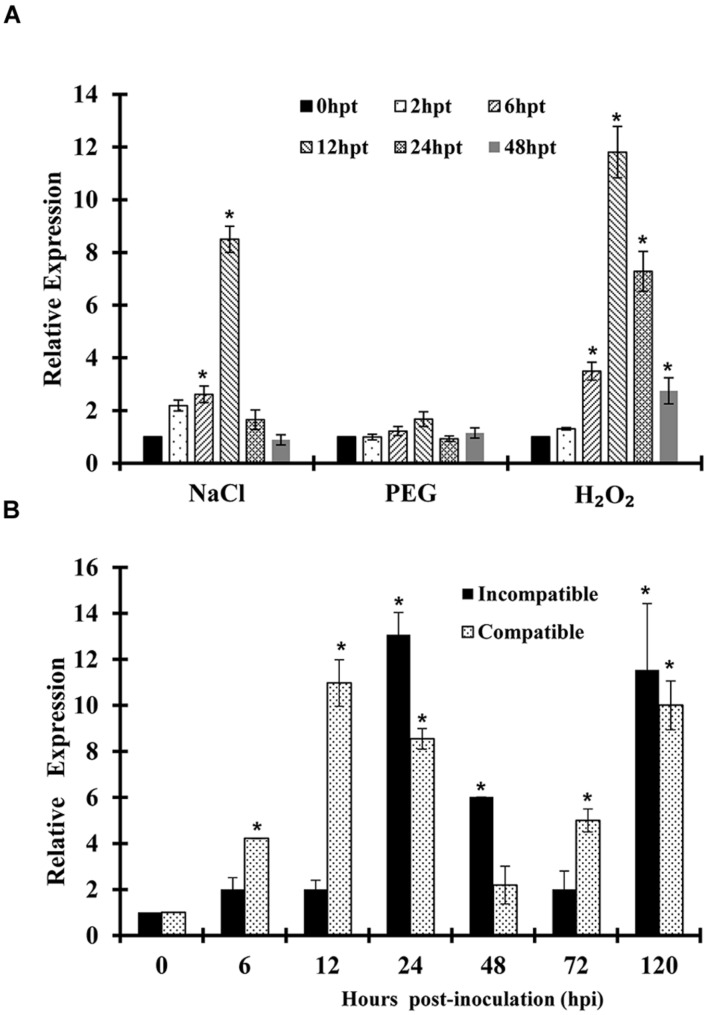
**Expression profiles of *TaSYP71* in response to abiotic and biotic stresses.** Expression levels were normalized to the wheat elongation factor *TaEF-1a* gene. The results are shown as the mean ± standard deviation of three biological replicates. **(A)** Wheat leaves were separately treated with PEG 6000, NaCl, or H_2_O_2_ and sampled at 0, 2, 6, 12, 24, 48 hpt. **(B)** Wheat leaves inoculated with CYR23 (incompatible) or CYR31 (compatible) were sampled at 0, 6, 12, 24, 48, 72, and 120 hpi. Asterisks indicate a significant difference (*p* < 0.05) from 0 hpi or hpt using Student’s *t*-test.

Quantitative real-time PCR (qRT-PCR) was used to determine the transcript profile of *TaSYP71*. For abiotic stresses, wheat seedlings were treated with polyethylene glycol 6000 (PEG 6000), NaCl, and H_2_O_2_. As shown in **Figure 3A**, following treatment by H_2_O_2_, expression levels of *TaSYP71* showed a significant upregulation from 6 h pthpt to 24 hpt and peaked at 12 hpt with an approximately 12-fold increase in expression. Following NaCl treatment, *TaSYP71* expression was dramatically elevated at 12 hpt with an approximately eightfold increase. However, *TaSYP71* did not show any significant induction by PEG 6000 treatment.

We also examined whether *TaSYP71* was induced by biotic stresses. During wheat and *Pst* interaction, *TaSYP71* was upregulated both in the compatible and incompatible interaction by 2- to 13-fold during infection. In the compatible interaction, the relative transcript level of *TaSYP71* was dramatically elevated at 12 hpi, followed by a slight decrease at 24 and 48 hpi and finally, a gradual increase at 72 and 120 hpi. The expression of *TaSYP71* in the incompatible interaction showed a similar pattern to the compatible interaction, except for the first peak at 24 hpi, which was later than the compatible interaction (**Figure 3B**).

### Overexpression of TaSYP71 in Fission Yeast Enhanced the Yeast Tolerance to H_2_O_2_ Stress

**FIGURE 4:**
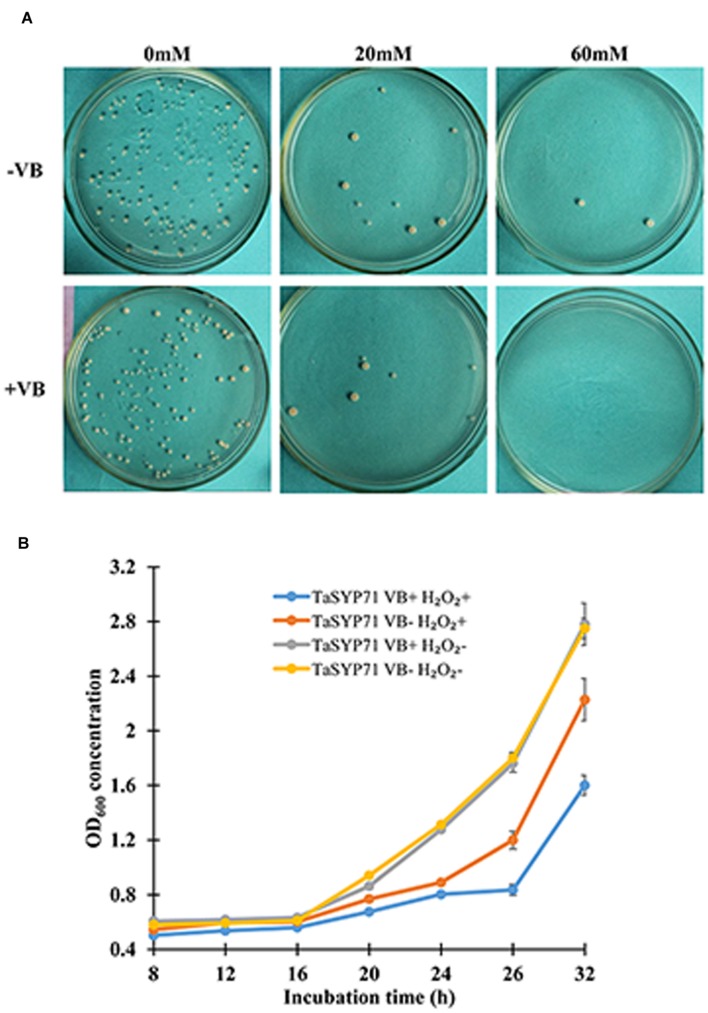
**Overexpression of TaSYP71 in fission yeast enhanced the ability of yeast to survive in H_2_O_2_ stress.** **(A)** Yeast cells expressing TaSYP71 were spotted on leucine dropout solid medium at the same concentration. The media contained 0, 20, and 60 mM H_2_O_2_ with (+VB) or without (-VB) thiamine. **(B)** Yeast cells carrying pREP3X-TaSYP71 were incubated in leucine dropout liquid medium with 20 mM H_2_O_2_, and the OD_600_ was measured at 12, 16, 20, 24, 28, and 32 h p-i.

To elucidate the exact role for TaSYP71 in response to H_2_O_2_ stress, we heterologously overexpressed TaSYP71 in *S. pombe*. The non-transformed yeast and empty vector pREP3X was used as a negative control. Yeast cells were cultured in medium with thiamine (repressing) or without thiamine (inducing) for 20 h. At the same H_2_O_2_ concentration (0 or 20 mM), the thiamine did not influence yeast growth in both controls (**Supplementary Table S3**). The number of colonies was reduced on the plate with 20 mM H_2_O_2_, which indicated that yeast growth, was inhibited by H_2_O_2_ treatment. No yeast cells survived when treated with 60 mM H_2_O_2_ in both controls. Moreover, yeast cells expressing pREP3X: eGFP showed fluorescence (**Supplementary Figure S2**) and the expression of eGFP was detected by western blot with anti-GFP antibody, which indicated that the system was feasible for protein expression. We used equal concentrations of yeast cells on the leucine dropout plates. As shown in **Figure 4A**, on H_2_O_2_ plate, the number of fission yeast cells expressing pREP3X: TaSYP71 in the absence of thiamine (- VB) was greater than the number of the control cells with thiamine (+ VB). Fission yeast cells survived even on the plate with a high concentration of H_2_O_2_ (60 mM). These results clearly demonstrated that overexpression of TaSYP71 in the fission yeast enhanced tolerance to H_2_O_2_.

The assay in leucine dropout liquid medium showed similar results. Regardless of the presence of thiamine, the OD_600_ of the group without H_2_O_2_ was much higher than that with H_2_O_2_. Without H_2_O_2_ treatment, there was no significant difference between yeast cells treated with thiamine and untreated cells. Following treatment with H_2_O_2_, fission yeast cells expressing TaSYP71 in the absence of thiamine (- VB) significantly increased (*p* < 0.05) compared to those in the presence of thiamine (+ VB) from 20 to 32 h after incubation (**Figure 4B**).

### *TaSYP71* Knockdown Wheat Plants Show Enhanced Susceptibility to *Pst*

**FIGURE 5:**
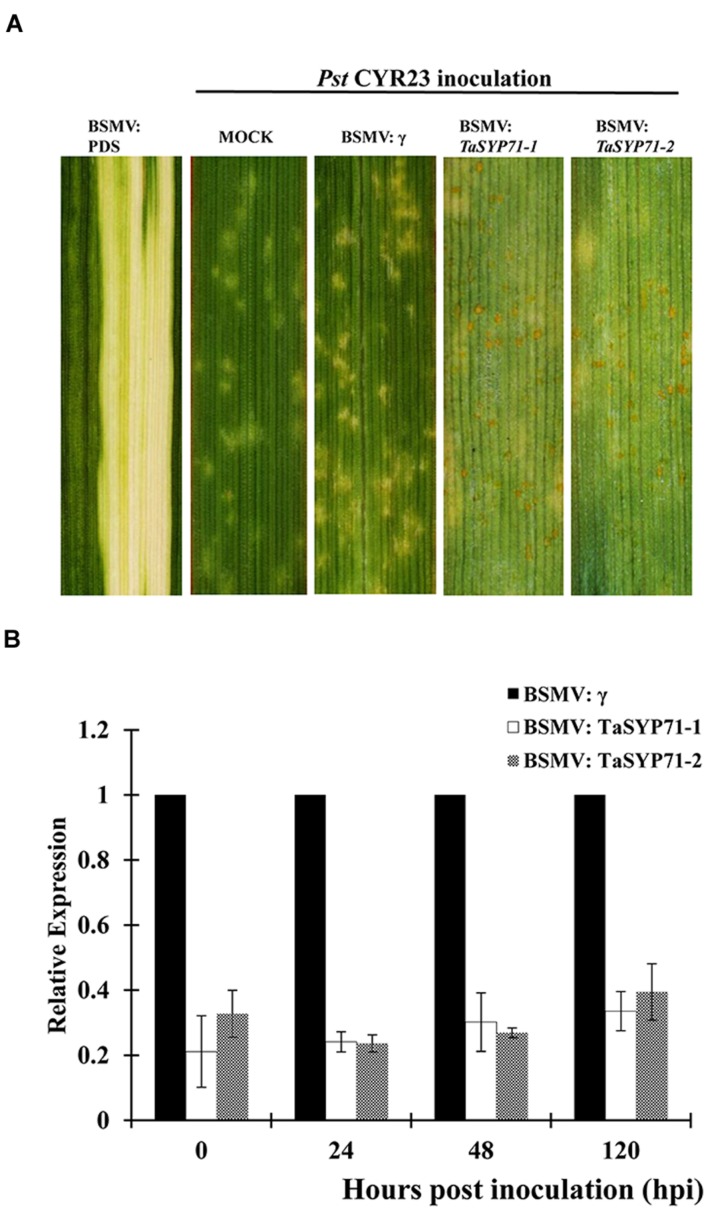
**Functional analyses of TaSYP71 during the incompatible interaction using a BSMV-mediated virus-induced gene silencing system.** **(A)** Photobleaching was evident on the fourth leaves of plants infected with BSMV: *TaPDS*. After inoculation of CYR23, disease phenotypes of the fourth leaves were assessed and are shown from the left: mock, pre-inoculated with BSMV: γ, BSMV: *TaSYP71*-1, and BSMV: *TaSYP71*-2. **(B)** Silencing efficiency assessment of the two segments of *TaSYP71* in the fourth leaves of *TaSYP71* knockdown plants compared with BSMV: γ during incompatible interaction. The data were normalized to the *TaEF-1a* expression level. The transcript levels of *TaSYP71* in BSMV: γ at each time point were set to one. Error bars represent variations among three independent replicates.

As *TaSYP71* was isolated from the wheat-*Pst* incompatible interaction, a VIGS system was applied to characterize the role of *TaSYP71* during the wheat-*Pst* incompatible interaction. None of the VIGS constructs was predicted to possess effective off-targets or cross silencing other SNARE transcripts in wheat as determined by the si-Fi software (**Supplementary Table S2**). BSMV: *TaPDS* (wheat phytoene desaturase gene) was used as a positive control for the gene silencing system. As shown in **Figure 5A**, photobleaching was presented in the fourth leaves of the BSMV: *TaPDS*-inoculated plants.

To determine the efficiency of silencing, qRT-PCR assays were performed on RNA samples extracted from the fourth leaves of wheat seedlings 0, 24, 48, and 120 hpi with the CYR23. These leaves were pre-infected with BSMV: γ, BSMV: *TaSYP71-1*, and BSMV: *TaSYP71-2*. Compared to the BSMV: γ control, the abundance of *TaSYP71* transcripts was greatly reduced to different extents (20–40%) in *TaSYP71* knockdown plants (**Figure 5B**).

Fourteen days after inoculation of CYR23, several necrotic spots were observed on the BSMV: γ and mock-inoculated wheat leaves. However, there were many necrotic spots and sporadic urediniospores on *TaSYP71*-knockdown plants. Resistance was greatly reduced in *TaSYP71*-knockdown wheat plants (shown in **Figure 5A**).

### Histological Changes of *Pst* Growth and Host Response

**FIGURE 6:**
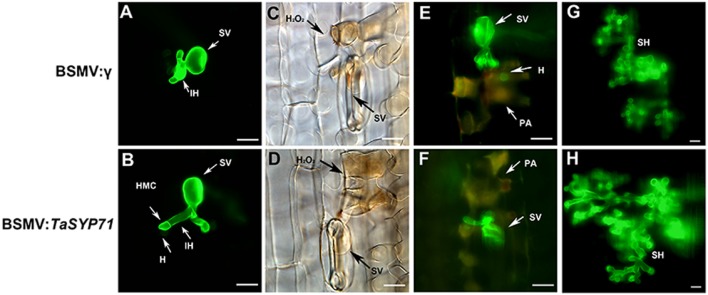
**Histological observation of fungal growth and host response during the incompatible interaction using a BSMV-mediated virus-induced gene silencing system.** *Pst* hypha were observed in leaf segments after staining by wheat germ agglutinin conjugated to the fluorophore Alexa-488. The fungal growth of *Pst* CYR23 in wheat leaves that were inoculated with BSMV: *γ*, BSMV:*TaSYP71* at 24 hpi **(A,B)**, 48 hpi **(E,F)** and 120 hpi **(G,H)** was observed under a fluorescence microscope. The accumulation of H_2_O_2_ at the infection site was observed in leaf segments sampled at 24 hpi after DAB staining **(C,D)**. SV, substomatal vesicle; IH, initial hyphae; PA, phenolic autofluorogens; H_2_O_2_, specific staining of H_2_O_2_ accumulation using DAB; HMC, haustorial mother cell; H, haustorium, SH, secondary hyphae. Bar = 20 μm.

To observe the histological changes associated with the enhanced susceptibility to *Pst* in these silenced plants, leaf segments from at least three plants inoculated with the CYR23 were harvested from each treatment. The phenolic autofluorogen accumulations per infection site in BSMV: *TaSYP71* pre-infected wheat leaves were significantly (*p* < 0.01) greater than the control at both 48 and 120 hpi (**Figure 6**; **Table 1**), indicating that knockdown of *TaSYP71* expression decreases plant defense reactions at infection sites. The H_2_O_2_ area stained by DAB was larger in *TaSYP71* knockdown plants than in the control 24 hpi of *Pst* (**Figure 6**; **Table 1**). The leaf samples were further stained using wheat germ agglutinin conjugated to the fluorophore Alexa-488 to facilitate rust hyphal observation. The *Pst* hyphal lengths in BSMV: *TaSYP71* pre-infected wheat leaves were significantly (*p* < 0.01) longer than those observed in BSMV: γ -infected leaves at 24 and 48 hpi (**Figure 6**; **Table 1**). The haustoria number of *TaSYP71* silenced plants were more than that in control plants (**Table 1**, *p* < 0.05). Meanwhile, the infection area 120 hpi was larger in *TaSYP71* knockdown plants (**Figure 6**; **Table 1**, *p* < 0.05). The hyphal branches showed no significant change following BSMV: *TaSYP71* treatment.

**Table 1:** Histological analysis of the incompatible interaction between wheat and *Pst* in *TaSYP71* knockdown plants.

Treatment	H_2_O_2_ area per infection site (μm^2^)	Necrotic area per infection site (μm^2^)		Hyphal length (μm)		Hyphal branches		Haustoria number		Infection area per infection site(μm^2^)
						
	24 hpi	48 hpi	120 hpi	24 hpi	48 hpi	24 hpi	48 hpi	24 hpi	48 hpi	120 hpi
BSMV: γ	249.7	690.2	822.5	15.5	17.5	1.8	2.26	1.26	1.53	16167.91
BSMV: *TaSYP71-1*	317.2^∗∗^	936.1^∗∗^	1107^∗^	18.6^∗∗^	21.7^∗∗^	1.9	2.38	1.57^∗^	1.83^∗^	23641.72^∗^
BSMV: *TaSYP71-2*	354.1^∗∗^	1046^∗∗^	1305^∗∗^	25.4^∗∗^	28.8^∗∗^	2.1^∗∗^	2.60	1.65^∗^	1.95^∗^	26909.49^∗^


*Wheat leaves were inoculated with BSMV: γ and two *TaSYP71* knockdown fragments, respectively, and then inoculated with CYR23. H_*2*_O_*2*_ area per infection site, necrotic cell area and the infection area were calculated by DP-BSW software. Hyphae length was measured from the junction of the substomatal vesicle and hypha to the tip of the hypha. All data were calculated from at least 50 infection sites. Significance was measured according to a paired sample t-test method. ^∗^*p* < 0.05; ^∗∗^*p* < 0.01.*

**FIGURE 7:**
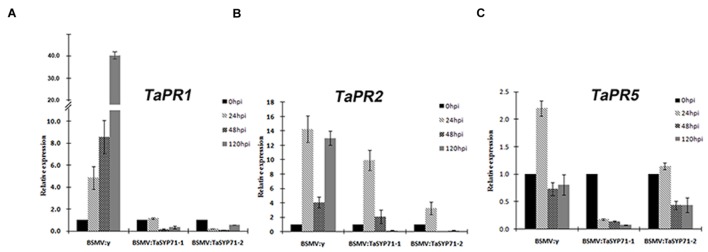
**The expression profiles of three pathogenesis-related genes *TaPR1***(A)**, *TaPR2***(B)**, and *TaPR5***(C)** were assessed 0, 24, 48, 120 h post *Pst* inoculation in *TaSYP71* knockdown plants in BSMV-inoculated plants.** The data were normalized to the wheat *TaEF-1a* expression level. The transcript levels of each gene at 0 hpi were set to one. Error bars represent variations among three independent replicates.

Furthermore, we examined the expression of defense related genes (*TaPR1*, *TaPR2* and *TaPR5*) in CYR23 inoculated *TaSYP71* knockdown wheat plants by qRT-PCR. The transcript level of *TaPR1* was gradually induced in BSMV: γ control wheat plants, however, expression was reduced or not changed in *TaSYP71* knockdown wheat plants. Although the expression of *TaPR2* was induced 24 hpi in *TaSYP71* knockdown, the induction intensity was lower than that in control wheat plants, and the transcript levels were sharply reduced 48 and 120 hpi (**Figure 7**). In control wheat plants, the expression of *TaPR5* was significantly induced 24 hpi. But the expression of *TaPR5* in *TaSYP71* knockdown wheat plants was reduced during *Pst* infection (**Figure 7**). Hence, we inferred that the expressions of *TaPRs* were reduced in *TaSYP71* knockdown plants challenged with the CYR23 compared to control, further suggesting that TaSYP71 is involved in the resistance of wheat to *Pst*.

### H_2_O_2_ Scavenging Gene Was Reduced by H_2_O_2_ Treatment in *TaSYP71* Knockdown Plants

**FIGURE 8:**
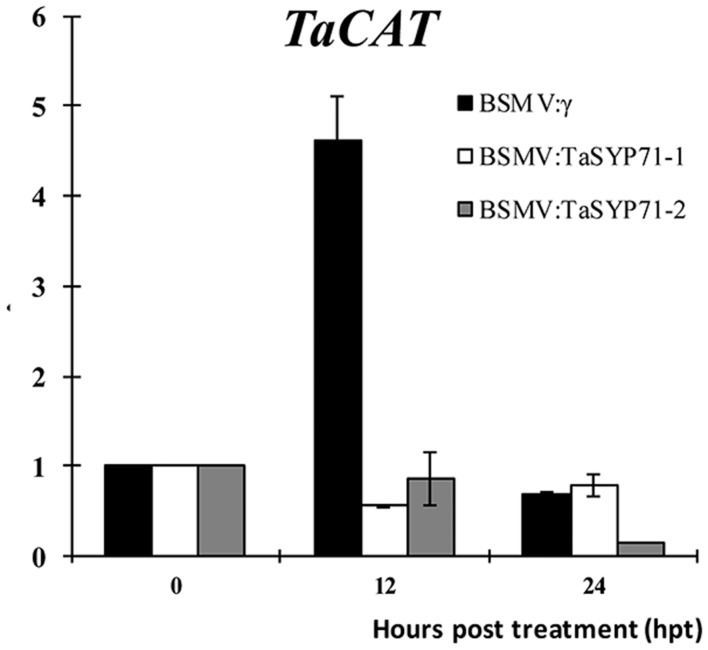
**The expression profiles of *TaCAT* was assessed 0, 12, and 24 h post 10 mM H_2_O_2_ treatment in *TaSYP71* knockdown plants.** The data were normalized to the wheat *TaEF-1a* expression level. The transcript levels of *TaCAT* at 0 hpt were set to one. Error bars represent variations among three independent replicates.

To test whether TaSYP71 involved in H_2_O_2_ scavenging, we assayed the transcript level of H_2_O_2_ scavenging related gene *TaCAT* in *TaSYP71* knockdown plants after H_2_O_2_ treatment. Compared with control plants that the expression of *TaCAT* was significantly induced 12 hpt, the transcript levels of *TaCAT* were almost unchanged or dropped a little in *TaSYP71* silenced plants (**Figure 8**), which partially uncovers that TaSYP71 is relevant to H_2_O_2_ scavenging.

## Discussion

SNARE proteins mediate intracellular vesicle fusion, which is an essential cellular process of eukaryotic cells. Along with membrane fusion, SNARE proteins also regulate many plant biological processes. In this study, from a wheat-*Pst* incompatible interaction cDNA library, we isolated a *SYP71* homolog, which has a Qc-SNARE domain and C-terminal transmembrane domain. However, this gene has no close relatives in other kingdoms; in other words, SYP71 is unique to the plant kingdom. Moreover, SYP71 was also found in the green algae *Chlamydomonas reinhardtii* and the moss *Physcomitrella patens*, suggesting that an essential role for plant-specific biological processes evolved early (Lipka et al., 2007). In *Arabidopsis*, SYP71 knockout mutant embryos and seedlings displayed strong morphological abnormalities (El Kasmi et al., 2013). Meanwhile, AtSYP71 also contributed to TuMV infection as reported previously (Wei et al., 2013). These indicate that SYP71 indeed contributes to diverse crucial biological processes. In this study, we focused on the involvement of wheat SYP71 protein in defense against biotrophic fungi. Three homologous were located in the A, B, and D genome, and the amino acid sequences of homoeoloci are conserved with only one residue difference (**Supplementary Figure S1**), which indicate that the function of these three copies might be similar and reduntant (Brenchley et al., 2012). Hence, it is necessary that all three copies will be silenced to further study their function.

In previous studies in *Arabidopsis*, AtSYP71 was localized to both the endoplasmic reticulum (ER) and the PM. When GFP-AtSYP71 was transiently expressed in the protoplasts of *Arabidopsis* suspension cultured cells, AtSYP71 was found to be localized to the ER (Uemura et al., 2004). However, AtSYP71 was mainly localized to the PM in transgenic *Arabidopsis* expressing GFP-AtSYP71 under its native promoter, although it was also localized to the ER in dividing regions (Suwastika et al., 2008). In our study, we showed that TaSYP71 was localized to the PM by transient expression of TaSYP71-eGFP in tobacco cells, suggesting that SYP71 might be involved in membrane trafficking to the PM. Fujiwara et al. (2014) employed immunoprecipitation and mass spectrometry and found that SYP71 interacts with Qa-SNARE SYP121, SYP122, and SYP132, which were located at the PM (Fujiwara et al., 2014). However, we cannot exclude the possibility of other location sites, which will need to be further analyzed in different physiological conditions.

An increasing body of evidence has suggested that SNARE genes are induced by various abiotic stresses. *NtSyp121* was strongly and transiently induced in tobacco leaves by ABA, drought, salt and wounding (Leyman et al., 1999). SYP61 has been reported to function in both salinity and osmotic stress tolerance as T-DNA mutant line *osm1* (*AtSYP61* disruption) exhibited increased sensitivity to both salinity and osmotic stress in a root-bending assay (Zhu et al., 2002). The expression pattern of *TaSYP71* in response to various abiotic stresses showed that SYP71 was induced by H_2_O_2_ and NaCl treatments; however, the expression pattern was unaffected when treated with PEG 6000, which may indicate that TaSYP71 is involved in salt and oxidative stress but not in drought stress. The expression pattern results support a similar but distinct function for TaSYP71 compared with other SNAREs. Because *TaSYP71* was induced following H_2_O_2_ treatment, we hypothesized a role for TaSYP71 in resistance to oxidative stress. To interpret the role of TaSYP71 in oxidative stress, we overexpressed TaSYP71 in fission yeast. Compared to the control, TaSYP71-overexpressing fission yeast showed more tolerance to H_2_O_2_ stress, which confirmed the role of TaSYP71 in oxidative stress. Furthermore, the assay for H_2_O_2_ treatment in *TaSYP71* silenced wheat plants indicates that TaSYP71 enhanced tolerance to H_2_O_2_ stress possibly by influencing the expression of *TaCAT* to remove the excessive H_2_O_2_ accumulation. One possibility is that SYP71 may enhance the ability of plants to remove ROS. Previous studies have confirmed that SNAREs could prevent H_2_O_2_-induced apoptosis (Levine et al., 2001). In our study, the transcript level of *TaSYP71* was peaked at 12 and 120 hpi in compatible interaction, representing the period of formation of haustoria and large amount of secondary hyphae respectively, which might indicate that stripe rust pathogen try to remove H_2_O_2_ accumulation by regulating the expression of *TaSYP71* to extend in its host. Meanwhile, the expression of *TaSYP71* was also induced during wheat infected by avirulent isolate CYR23, possibly suggesting its role in wheat resistance to *Pst* attack. Taken together, we inferred that vesicles containing H_2_O_2_ may fuse with the PM easily and exhaust outside of the plant cells to relieve the damage to the cells, and during the wheat-*Pst* interactions, ROS is key arm for pathogen or host to survive. However, the enigma that how TaSYP71 performs its role still needs to be excavated.

Accumulating evidence has indicated that plant endo- and exocytotic processes play crucial roles in plant-microbe interactions (Lipka et al., 2007). SYP71 was reported to participate in plant defense against various pathogens. The SNARE protein Syp71 is essential for turnip mosaic virus infection by mediating fusion of virus-induced vesicles with chloroplasts. OsSYP71-overexpressing transgenic lines grew better than wild type plants, and the expression of PR-1b and peroxidase was significantly enhanced after rice blast inoculation, which demonstrated that OsSYP71 confers resistance to rice blast (Bao et al., 2012). However, there has been limited evidence for the role of SYP71 in plant defense against biotrophic fungi. In this study, to determine the role of TaSYP71 in wheat-*Pst* interactions, the expression of *TaSYP71* was suppressed by a BSMV-mediated VIGS system. *TaSYP71* knockdown plants were more susceptible, the hyphal length was longer, the infection area was larger and more haustoria were formed in *TaSYP71* knockdown plants compared with control plants. Additionally, the expression of PR genes was down-regulated in *TaSYP71* silenced wheat plants. These results reported here indicate that TaSYP71 participates in wheat defense against *Pst* infection.

Surprisingly, in *TaSYP71* knockdown plants, the necrotic area per infection site was increased compared with that in control plants associated to higher infection areas. One possibility is that when *TaSYP71* was suppressed, H_2_O_2_ containing vesicle trafficing was tardy and inefficient, which lead to a later cell death insufficient to restrict fungal infection. Of course, we could not exclude the possibility that there are other redundant apoptotic factors that function in the interaction. In addition, we found that the accumulation of H_2_O_2_ was enhanced in *TaSYP71* knockdown plants compared to control 24 hpi, which was the peak period of oxidative bursts as shown by previous histological and cytological observations. At optimal concentrations, H_2_O_2_ acts as a second messenger for the induction of defense genes, but excessive accumulation of ROS is detrimental to plants. Considering the role of TaSYP71 in H_2_O_2_ stress, we speculated that TaSYP71 might regulate the H_2_O_2_ balance in host cells and H_2_O_2-_induced signaling pathways, thereby contributing to *Pst* resistance. Localized at the PM, TaSYP71 facilitates signaling H_2_O_2_ -containing vesicles moving from host cells to extracellular regions, thus conferring *Pst* resistance.

In sum, we speculate that there are correlations between enhanced H_2_O_2_ tolerance and *Pst* resistance. This may be related to vesicle fusion and trafficking. However, the regulation of TaSYP71 in wheat under *Pst* stresses may be more complex than currently understood. Our study confirmed the positive role of TaSYP71 in host defense against *Pst*. Stable transgenic SNARE RNAi wheat should be used for further studies to explore the precise mechanism of how TaSYP71 modulates host defense.

## Author Contributions

ML, HL, XW, and ZK designed the experiment. ML, YP, and HL performed the experiments and analyzed the data. XW and LD helped with data interpretation and article editing. ML wrote the manuscript.

## Conflict of Interest Statement

The authors declare that the research was conducted in the absence of any commercial or financial relationships that could be construed as a potential conflict of interest.

## Footnotes

1 http://wheat-urgi.versailles.inra.fr/Seq-Repository/BLAST

## Supplementary Material

**FIGURE S1 | Multi-sequence alignment of *TaSYP71* copies.** The sequence of 1AL is the sequence we obtained. **(A)** Multi-alignment of the mRNA sequence of the three copies of *TaSYP71*. Black underline indicates 5′ and 3′ UTR region as shown in the figure. **(B)** Alignment of amino acid sequence of the two copies of *TaSYP71* (on chromosome 1A and 1B).

**FIGURE S2 | Over-expression of TaSYP71 in fission yeast cells.** The fluorescence of fission yeast cells carrying pREP3X-eGFP was observed at Olympus DP71 fluorescence microscope **(A)** and the corresponding cell morphology was observed under bright field **(B)**. Western blot analysis of eGFP expression levels in yeast cells with anti-GFP antibody **(C)**.
