# Supplementary figures and images for: TaSYP71, a Qc-SNARE, Contributes to Wheat Resistance against Puccinia striiformis f. sp. tritici
Source: Front Plant Sci. 2016 Apr 21;7:544. doi: 10.3389/fpls.2016.00544 (PMC4838636; doi:10.3389/fpls.2016.00544)

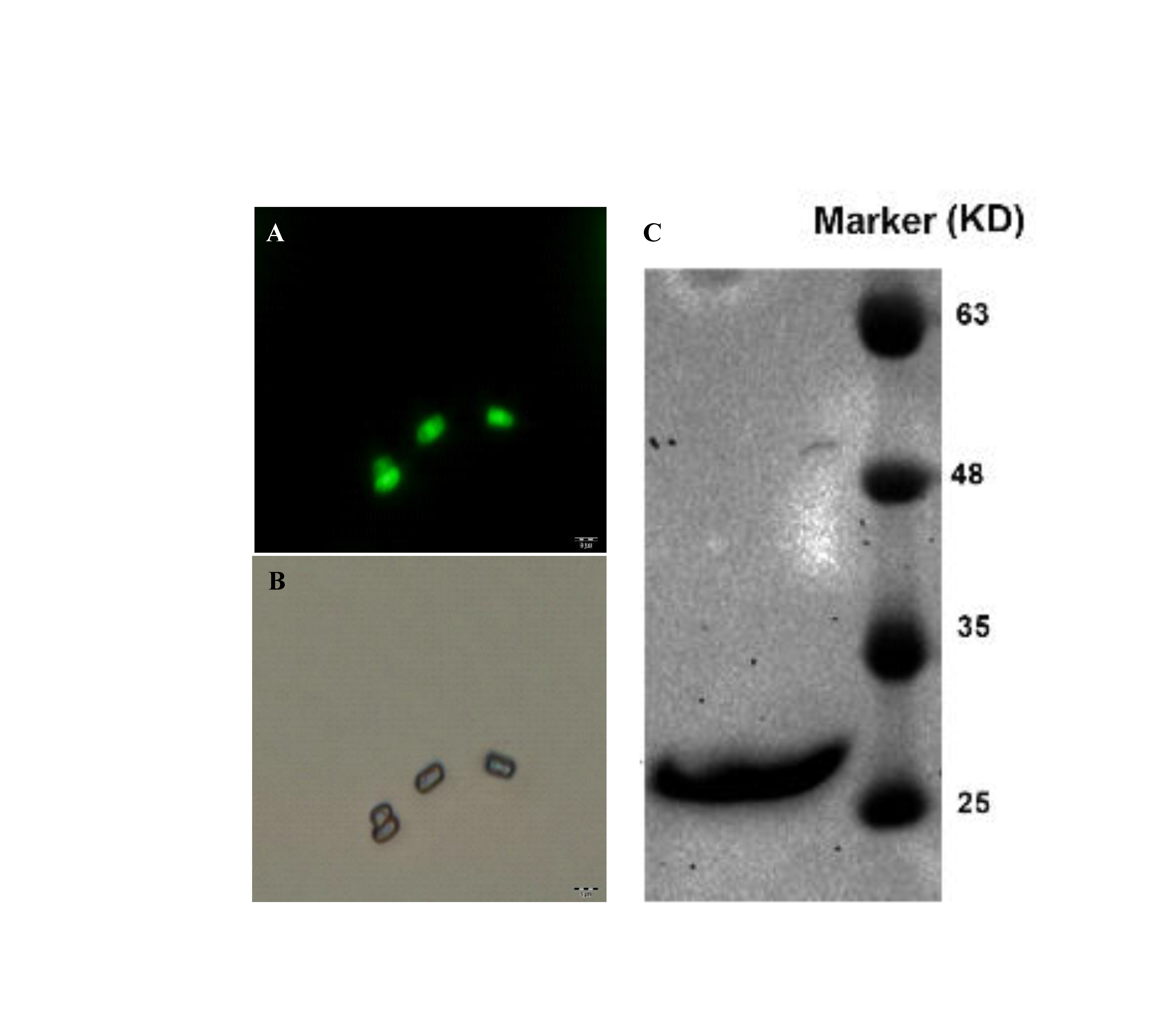

Supplement: Supplementary file 2 [file Image_2.TIFF]
