# Supplementary material for: TaSYP71, a Qc-SNARE, Contributes to Wheat Resistance against Puccinia striiformis f. sp. tritici
Source: Front Plant Sci. 2016 Apr 21;7:544. doi: 10.3389/fpls.2016.00544 (PMC4838636; doi:10.3389/fpls.2016.00544)
Supplement: Supplementary file 3 [file Table_1.DOCX]

**Supplementary Table 1. Primers used in this study**

| **Primer Name** | **Primer 5’-3’** | **Purpose** |
| --- | --- | --- |
| TaSYP71-qRT-F | TTGGATGTTATTGGTGAA | qRT-PCR for validation of the expression levels of *TaSYP71* |
| TaSYP71-qRT-R | CGTTGGTATTCTTCAGAT | qRT-PCR for validation of the expression levels of *TaSYP71* |
| TaSYP71-1302-F | GGACTAGTTGACGGTGATCGACATCCT | Clone TaSYP71 into pCambia1302 for subcellular localization analysis |
| TaSYP71-1302-R | TTCCTAGGCTTCTTAAGCACATTGTAAAGGTAG | Clone TaSYP71 into pCambia1302 for subcellular localization analysis |
| TaSYP71-VIGS1-F | CCTTAATTAAGTCGGAGCGGGCGGAGA | Clone specific segment of TaSYP71 to the BSMV vector for silencing |
| TaSYP71-VIGS1-R | ATAAGAATGCGGCCGCGAGGCGGGCGAAGGGGT | Clone specific segment of TaSYP71 to the BSMV vector for silencing |
| TaSYP71-VIGS2-F | CCTTAATTAAATTATCCTGCTCTGTATC | Clone specific segment of TaSYP71 to the BSMV vector for silencing |
| TaSYP71-VIGS2-R | ATAAGAATGCGGCCGCCAAGTAGAGAATCCAATG | Clone specific segment of TaSYP71 to the BSMV vector for silencing |
| TaSYP71-pREP3x-F | CGGGATCCATGACGGTGATCGACATCCT | Clone TaSYP71 into pREP3x for overexpression analysis |
| TaSYP71-pREP3x-R | TCCCCCGGGTCACTTCTTAAGCACATTGTAAAGGT | Clone TaSYP71 into pREP3x for overexpression analysis |
| TaEF-qRT-F | TGGTGTCATCAAGCCTGGTATGGT | The wheat endogenous reference for normalization in qRT-PCR |
| TaEF-qRT-R | ACTCATGGTGCATCTCAACGGACT | The wheat endogenous reference for normalization in qRT-PCR |
| TaPR1-qRT-F | GAGAATGCAGACGCCCAAGC | qRT-PCR for validation of the expression levels of *TaPR1* |
| TaPR1-qRT-R | CTGGAGCTTGCAGTCGTTGATC | qRT-PCR for validation of the expression levels of *TaPR1* |
| TaPR2-qRT-F | AGGATGTTGCTTCCATGTTTGCCG | qRT-PCR for validation of the expression levels of *TaPR2* |
| TaPR2-qRT-R | AAGTAGATGCGCATGCCGTTGATG | qRT-PCR for validation of the expression levels of *TaPR2* |
| TaPR5-qRT-F | CAAGCAGTGGTATCAACGCAGAG | qRT-PCR for validation of the expression levels of *TaPR5* |
| TaPR5-qRT-R | GTGAAGCCACAGTTGTTCTTGATGTT | qRT-PCR for validation of the expression levels of *TaPR5* |
| TaCAT-qRT-F | TGCCTGTGTTTTTTATCCGAGA | qRT-PCR for validation of the expression levels of *TaCAT* |
| TaCAT-qRT-R | CTGCTGATTAAGGTGTAGGTGTTGA | qRT-PCR for validation of the expression levels of *TaCAT* |
