# Supplementary material for: TaSYP71, a Qc-SNARE, Contributes to Wheat Resistance against Puccinia striiformis f. sp. tritici
Source: Front Plant Sci. 2016 Apr 21;7:544. doi: 10.3389/fpls.2016.00544 (PMC4838636; doi:10.3389/fpls.2016.00544)
Supplement: Supplementary file 4 [file Table_2.DOCX]

**Table S2.** Prediction of *TaSYP71*-VIGS construct off-target transcripts ^a^.

|  | Database ID ^b^ | All hits | Efﬁcient hits | Blast Annotation |
| --- | --- | --- | --- | --- |
| *TaSYP71-1* |  | 0 | 0 |  |
| *TaSYP71-2* | Ta#S61829719 | 22 | 14 | Syntaxin-71 |

^a^ Simulations were run using Si-Fi software (v3.2) for off-target prediction (http://labtools.ipk-gatersleben.de).

^b^ Wheat gene sequence files were obtained from the wheat UniGene set downloading from ftp://ftp.ncbi.nih.gov/repository/UniGene/Triticum_aestivum/.
