# Supplementary material for: TaSYP71, a Qc-SNARE, Contributes to Wheat Resistance against Puccinia striiformis f. sp. tritici
Source: Front Plant Sci. 2016 Apr 21;7:544. doi: 10.3389/fpls.2016.00544 (PMC4838636; doi:10.3389/fpls.2016.00544)
Supplement: Supplementary file 5 [file Table_3.DOCX]

Table S3. Colony number of fission yeast on solid medium plate with different treatment.

| Treatment^a^ | 0 mM^b^ | | 20 mM | |
| --- | --- | --- | --- | --- |
|  | -VB | +VB | -VB | +VB |
| *S.pombe* | 140.67 | 136.33 | 7.22 | 6.89 |
| *S.pombe*: pREP3x | 131.11 | 122.44 | 7.33 | 7.44 |

^a^ Three independent replicates were conducted and in each independent replicate three mechanical replicates were performed.

Significance was measured according to a paired sample t-test method.

^b^ Yeast cell were diluted 5 times in 0 mM H_2_O_2_ treatment.

None yeast cell was survived in solid medium with 60 mM H_2_O_2._
